# Design, Development, Physicochemical Characterization, and In Vitro Drug Release of Formoterol PEGylated PLGA Polymeric Nanoparticles

**DOI:** 10.3390/pharmaceutics14030638

**Published:** 2022-03-14

**Authors:** Ernest L. Vallorz, David Encinas-Basurto, Rick G. Schnellmann, Heidi M. Mansour

**Affiliations:** 1 Skaggs Pharmaceutical Sciences Center, The University of Arizona R. Ken Coit College of Pharmacy, 1703 E Mabel St., Tucson, AZ 85721, USA; vallorz@pharmacy.arizona.edu (E.L.V.); dencinas@pharmacy.arizona.edu (D.E.-B.); schnell@pharmacy.arizona.edu (R.G.S.); 2 Department of Medicine, The University of Arizona College of Medicine, 501 N Campbell Ave., Tucson, AZ 85724, USA; 3 BIO5 Institute, The University of Arizona, 1657 E Helen St., Tucson, AZ 85719, USA; 4 Center for Translational Science, Florida International University, Port St. Lucie, FL 34987, USA

**Keywords:** nanoparticle, solid-state characterization, in vitro, drug release kinetics modeling, PEGylation, PLGA diblock copolymer, biodegradable, biocompatible, amine, emulsion, polyvinyl alcohol (PVA), Pluronic triblock copolymer, trehalose, sucrose

## Abstract

Polymeric nanoparticles’ drug delivery systems represent a promising platform for targeted controlled release since they are capable of improving the bioavailability and tissue localization of drugs compared to traditional means of administration. Investigation of key parameters of nanoparticle preparation and their impact on performance, such as size, drug loading, and sustained release, is critical to understanding the synthesis parameters surrounding a given nanoparticle formulation. This comprehensive and systematic study reports for the first time and focuses on the development and characterization of formoterol polymeric nanoparticles that have potential application in a variety of acute and chronic diseases. Nanoparticles were prepared by a variety of solvent emulsion methods with varying modifications to the polymer and emulsion system with the aim of increasing drug loading and tuning particle size for renal localization and drug delivery. Maximal drug loading was achieved by amine modification of polyethylene glycol (PEG) conjugated to the poly(lactic-co-glycolic acid) (PLGA) backbone. The resulting formoterol PEGylated PLGA polymeric nanoparticles were successfully lyophilized without compromising size distribution by using either sucrose or trehalose as cryoprotectants. The physicochemical characteristics of the nanoparticles were examined comprehensively, including surface morphology, solid-state transitions, crystallinity, and residual water content. In vitro formoterol drug release characteristics from the PEGylated PLGA polymeric nanoparticles were also investigated as a function of both polymer and emulsion parameter selection, and release kinetics modeling was successfully applied.

## 1. Introduction

Developments in the formulation and application of nanotechnology have advanced the administration of drugs to different organs for a wide array of diseases. Compared to carrier-independent conventional dosage forms, drug nanoparticles possess many key advantages. Nanoparticle drug delivery allows for improved solubility and stability of the drug, sustained drug release, improved patient compliance, and targeted delivery that can increase the therapeutic index of medicines [1,2,3].

Polymer-based particles are frequently studied and used as drug carriers in a wide variety of therapeutic applications since controlling their synthesis enables their physicochemical properties and drug release properties to be customized [4]. Thus, size distribution or side-chain composition of the polymer can be modified to improve nanoparticle performance. Among various polymers, the most widely used are aliphatic polyesters such as poly(lactic-co-glycolic acid) (PLGA) due to this particular polyester’s favorable biodegradation characteristics and biocompatibility, and its success in FDA-approved sustained release injectable marketed pharmaceutical products [5]. Advances in the design of these particles has improved stability and circulation through PEGylation [6,7,8], biospecific targeting [9,10,11,12], and improved drug loading [2,13].

Formoterol fumarate dihydrate, a long-acting β_2_ agonist (LABA), is a United States Food and Drug Administration (FDA)-approved agent for the treatment of asthma and obstructive pulmonary disease via inhalation. More recently, formoterol fumarate dihydrate has shown promise in treating mitochondrial dysfunction, which occurs in a variety of acute and chronic injuries [14,15,16,17,18,19,20,21]. Given that the β_2_-adrenergic receptor is ubiquitously expressed, systemic delivery of formoterol risks potentially toxic side effects, particularly due to acute cardiovascular effects such as tachycardia and hypotension [22,23,24,25], as well as long-term cardiac remodeling [26,27,28,29]. Polymeric nanoparticles targeting the renal proximal tubules have shown promise at avoiding potential systemic toxicity using the FDA-approved diblock copolymer poly(lactic-co-glycolic) acid (PLGA) conjugated with FDA-approved polyethylene glycol (PEG) [10,30,31,32]. Thus, combining the potential renal targeting of polymeric nanoparticles with the mitochondrial biogenic and renoprotective effects of formoterol is likely to allow for enhanced biogenic effects and potentially improved recovery following renal injury while minimizing toxicity. In a proof-of-concept study, our laboratory has recently shown an ability to successfully deliver formoterol to the kidneys, providing renal drug targeting and sustained renal mitochondrial biogenesis while reducing the effect of the drug on the heart [33]. The purpose of this comprehensive and systematic study was to improve upon the previously synthesized nanoparticles, which were limited by formoterol drug loading and rapid release, by modifying the route of nanoparticle synthesis and through an evaluation of polymer modifications. Additionally, this study will characterize the physicochemical properties of the nanoparticles, quantify sustained release behavior, correlate the physicochemical properties with the sustained drug release properties, and mathematically model drug release with known mechanistic drug release models. To the authors’ knowledge, this is the first study to report these findings.

## 2. Materials and Methods

### 2.1. Materials

Formoterol fumarate dihydrate [C_19_H_24_N_2_O_4_·0.5C_4_H_4_O_4_·H_2_O; 420.46 g/mol; 99.8% pure] was purchased from APAC Pharmaceuticals (Columbia, MD, USA). Poly(ethylene glycol) methyl ether-block-poly(lactide-co-glycolide) (PLGA-PEG), lactide:glycolide ratio 50:50, PLGA average M_n_ 55,000 g/mol, 30,000 g/mol, and 15,000 g/mol; PEG average M_n_ 5000 g/mol was purchased from Sigma-Aldrich (St. Louis, MO, USA). Poly(lactide-co-glycolide) methyl ether block- poly(ethylene glycol)-amine (PLGA-PEG-HN_2_) PLGA average M_n_ 20,000 g/mol, PEG average M_n_ 5000 g/mol and poly(lactide-co-glycolide) methyl ether block- poly(ethylene glycol)-carboxylic acid (PLGA-PEG-COOH) PLGA average M_n_ 20,000 g/mol, and PEG average M_n_ 5000 g/mol were purchased from Nanosoft Polymers (Winston-Salem, NC, USA). Acetic acid (HPLC grade) was purchased from ThermoFisher (Waltham, MA, USA). Ethanol (99.9% purity, HPLC grade), hydrochloric acid 1 N (ACS grade), sodium hydroxide 1 N (ACS grade), poly(vinyl alcohol) (PVA) (molecular weight 89,000–98,000 g/mol, >99% hydrolyzed, reagent grade), Pluronic F127™ (molecular weight~12,600 g/mol, reagent grade), sodium choate hydrate (>99% purity), sodium deoxycholate (>99% purity) potassium phosphate monobasic (>99% purity), potassium phosphate dibasic (>98% purity), formic acid (97.5–98.5% purity), d-mannitol (ACS grade), and sucrose (99.5% purity) were purchased from Sigma-Aldrich (St. Louis, MO, USA). (+)-Trehalose dihydrate (387.32 g/mol) was purchased from Acros Organics (Fair Lawn, NJ, USA). Choloroform (ACS grade), anhydrous acetonitrile (LCMS grade), and methanol (LCMS grade) were purchased from Spectrum Chemical Mfg. Corp. (Gardena, CA, USA). 0.45 µm polyvinylidene fluoride (PVDF) and 0.45 µm polytetrafluoroethylene (PTFE) membrane filters were purchased from MilliporeSigma (Burlington, MA, USA).

### 2.2. Methods

#### 2.2.1. Solubility of Formoterol Fumarate Dihydrate in Aqueous and Organic Media

The solubility of formoterol fumarate dihydrate (APAC Pharmaceuticals, Columbia, MD, USA) was determined in various common aqueous and organic media to determine their suitability for use in the preparation of nanoparticles. The excess of formoterol fumarate dihydrate was added to a known volume of solvent. For variable pH samples, solution pH was adjusted with either hydrochloric acid or sodium hydroxide solutions (1 M Sigma-Aldrich, St. Louis, MO, USA). Vials were rotated gently for 24 h at 25 °C, as previously described [34]. Test solutions were filtered using 0.45 µm PVDF or PTFE membrane filters (MilliporeSigma, Burlington, MA, USA) for aqueous and organic solvents, respectively. Quantitative analysis of formoterol content was determined by high performance liquid chromatography (HPLC), as previously described [35]. Briefly, a C18- column (4.6 mm × 250 mm length, 5 µm pore size) (Phenomenex, Torrance, CA, USA) mobile phase consisting of methanol (Spectrum Chemical MFG Corp., Gardena, CA, USA) and 50 mM phosphoric acid (Sigma-Aldrich, St. Louis, MO, USA) buffer with 1% acetic acid (ThermoFisher, Waltham, MA, USA) at a ratio of 65:35, 1.0 mL/min flow rate and a column temperature of 40 °C was used to quantify formoterol content.

#### 2.2.2. Preparation of Polymeric Nanoparticles

Nanoparticles were prepared by either single or double emulsion methods. For nanoparticles prepared by oil-in-water single emulsion, the polymer was dissolved in chloroform and formoterol fumarate dihydrate were dissolved in methanol before being added to the polymer solution. This was then emulsified in an aqueous solution of 3% PVA (Sigma-Aldrich, St. Louis, MO, USA) using a microtip probe sonicator (Qsonica, Newton, CT, USA) at 60 Watt of energy output for 3 min over ice and the organic solvent allowed to evaporate with stirring (700 rpm) at room temperature for at least 8 h. For nanoparticles prepared by water-in-oil-in-water double emulsion, formoterol fumarate dihydrate was dissolved in aqueous media before being added to a solution of polymer in chloroform (Spectrum Chemical Mfg. Corp., Gardena, CA, USA) at a 1:2 aqueous:organic ratio. An initial emulsion was formed by sonicating at 60 Watt using a microtip probe sonicator for 30 s before being added to a 3% PVA solution and sonicated again. The organic solvent was then allowed to evaporate with stirring at room temperature. For all nanoparticle syntheses, the particles were collected by centrifugation at 15,000 relative centrifugal force (rcf) and washed three times with distilled ultrapure water (18.2 MΩ.cm) (Milli-Q Plus, MilliporeSigma, Burlington, MA, USA). The samples were lyophilized at −80 °C under a vacuum < 0.133 mmHg (FreeZone 4.5 L, Labconco, Kansas City, MO, USA) with or without cryoprotectant and stored at −20 °C until use.

#### 2.2.3. Effect of Polymeric Nanoparticle Synthesis Parameters on Formoterol Drug Loading

To determine drug loading, nanoparticles were dissolved in an acetonitrile solution and analyzed by HPLC method reported above. Drug loading was calculated, as previously reported [36], using Equation (1):

(1) $$\mathrm{DL}\;(\%)=\frac{the\;amount\;of\;formoterol\;assayed}{the\;total\;amount\;of\;nanoparticles\;in\;the\;preparation}\times 100$$

#### 2.2.4. Effect of Polymeric Nanoparticle Synthesis Parameters on Particle Size

Nanoparticle size was determined immediately following washing. For the evaluation of the lyophilized particles, approximately 1 mg of nanoparticle was suspended in ultrapure water and centrifuged for 10 min at 15,000 rcf to remove the cryoprotectant. Nanoparticles were suspended at a concentration of ~1 mg/mL with ultrapure water, and hydrodynamic particle size was determined by photon correlation spectroscopy using the Zetasizer Nano ZS (Malvern Instruments Ltd., Malvern, UK) under previously reported conditions [37]. The suspended nanoparticles were evaluated using a scattering angle of 173° at a temperature of 25 °C in triplicate with a minimum of 10 measurements taken per replicate.

#### 2.2.5. Impact of Nanoparticle Synthesis Parameters on Zeta Potential

Nanoparticle zeta potential (ζ) measurements were carried out in 0.1× normal saline solution at 25 °C and pH 7.2. The mean ζ was determined using the Zetasizer Nano ZS (Malvern Instruments Ltd., Malvern, UK) phase analysis light scattering technique.

#### 2.2.6. Impact of Nanoparticle Synthesis Parameters on In Vitro Drug Release

Nanoparticles prepared as described above were dispersed in 10 mL of phosphate buffered saline (PBS) (pH 7.4) and incubated at 37 °C with gentle stirring, as previously described [38]. At determined intervals, an aliquot was taken and centrifuged at 15,000 rcf for 10 min. The supernatant was extracted and replaced with an equal volume of fresh PBS in order to maintain sink conditions. Formoterol content was chemically analyzed and quantified by HPLC, as described above. Modeling of formoterol in vitro drug release was carried out for three kinetic models, namely, the zero-order, first-order, and Korsmeyer–Peppas models. Zero-order kinetics were fitted to Equation (2):

(2) $$Q_{t}-Q_{0}=k_{0}t$$

where *Q_t_* is the amount of drug released after time *t*, *Q*_0_ is the initial amount of drug in solution, and *k*_0_ is the zero-order rate constant. First-order kinetics were fit to Equation (3):

(3) $$\ln Q_{t}=\ln Q_{0}-k_{1}t$$

where *Q_t_* is the amount of drug released after time *t*, *Q*_0_ is the initial amount of drug in solution, and *k*_1_ is the first-order rate constant. Finally, release kinetics were fit to the Korsmeyer–Peppas model, Equation (4):

(4) $$Q_{t}=kt^{n}$$

where *Q_t_* is the amount of drug released after time *t*, *k* is the rate constant, and *n* is the diffusion exponent for drug release. Nanoparticle release was determined in triplicated (*n* = 3) for each preparation. Data were plotted using Prism 9.0 (GraphPad^®^ Software, San Diego, CA, USA).

#### 2.2.7. Characterization of Nanoparticle Surface Morphology

Nanoparticle size and surface morphology was visualized using SEM (FEI Inspect S SEM, FEI Company, Hillsboro, OR, USA). Powders were deposited on double-sided carbon conductive adhesive tabs (Ted-Pella, Inc., Redding, CA, USA) attached to aluminum SEM stubs (Ted-Pella, Inc., Redding, CA, USA) and sputter-coated (Anatech Hummer 6.2, Union City, CA, USA) with gold for 90 s under argon plasma as previously reported [39].

#### 2.2.8. X-Ray Powder Diffraction (XRPD)

The crystallinity of PLGA-PEG-NH_2_, formoterol fumarate dihydrate, sucrose, trehalose, and lyophilized nanoparticles with and without cryoprotectant were examined using XRPD. The diffraction patterns of the samples were collected at room temperature scanning between 5.0° and 70.0° (2θ) at a rate of 2.00° per minute using a Philips PANalytical X’Pert PRO MPD (Malvern Panalytical, Malvern, UK) equipped with copper X-ray source (Kα radiation with λ = 1.5406 Å). The samples were loaded onto zero background single crystal silicon holders, as previously reported [37,39].

#### 2.2.9. Thermal Analysis of Lyophilized Nanoparticles

Thermal analysis was performed by differential scanning calorimetry (DSC) and cross-polarized hot stage microscopy (HSM). DSC analysis was conducted as previously reported [40,41]. Thermal analysis and phase transition measurements for raw formoterol fumarate dihydrate, raw PLGA-PEG-NH_2_ (20,000 MW PLGA, 5000 MW PEG), raw sucrose, raw trehalose, and lyophilized PLGA-PEG-NH_2_ used with or without either sucrose or trehalose as a cryoprotectant were studied. Thermograms were acquired using the TA Q1000 differential scanning calorimeter with RSC090 colling accessory (TA Instruments, New Castle, DE, USA). A mass of between 1 and 5 mg of sample was weighed into anodized aluminum hermetic pans (TA Instruments) with an empty pan used as a reference. DSC measurements were performed at a heating rate of 10 °C/min from 0 to 350 °C. Ultrapure nitrogen gas was used as the purging gas at a rate of 50 mL/min. Analysis of thermograms was conducted using TA Universal Analysis (TA Instruments). All measurements were carried out in triplicate.

Solid-state phase transitions of the lyophilized nanoparticles were observed using cross-polarized light HSM similarly to previously reported [39]. Microscopy was conducted using a Leica DMLP cross-polarized microscope (Leica Mircosystems, Wetzlar, Germany) equipped with a Mettler FP 80 central processor and FP82 hot stage (Mettler Toldeo, Columbus, OH, USA). Lyophilized particles were mounted on a microscope slide and heated at a rate of 10 °C/min from 25 °C to 300 °C. The images were digitally captured using a Nikon Coolpix 8800 digital camera (Nikon, Tokyo, Japan) under 100× total magnification.

#### 2.2.10. Residual Water Content Analysis by Karl Fischer Titration

The residual water content of lyophilized nanoparticles was quantified by Karl Fischer titration (KFT) colorimetric assay using a TitroLine^®^ 7500 trace titrator (SI Analytics, Mainz, Germany). Around 3–7 mg of the sample was dissolved in 5 mL AQUA STAR anhydrous acetonitrile and injected into the titration cell. The measured moisture content was expressed in percentage as the result of the KFT. All measurements were completed in triplicate. 

#### 2.2.11. Statistical Analysis

Comparison of the difference between three groups was performed by one-way analysis of variance (ANOVA) with Tukey’s *post hoc* test for comparisons (Prism 9.0, GraphPad Software, San Diego, CA, USA). In all cases, the *p* values of 0.05 or less were considered significant.

## 3. Results

### 3.1. Solubility of Formoterol Fumarate Dihydrate in Aqueous and Organic Media

**Figure 1:**
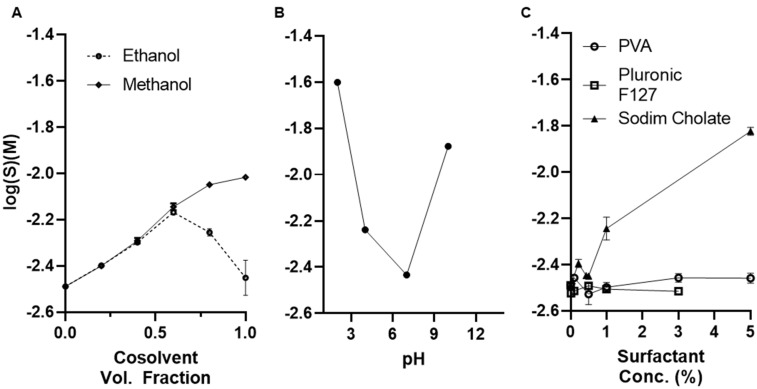
Solubility of formoterol determined in (**A**) ethanol and methanol, (**B**) water at various pH, and (**C**) aqueous solutions of various surfactants at 25 °C and gentle shaking for 24 h. Data presented are mean (*n* = 3) ± s.d.

The solubility of formoterol and its salt formoterol fumarate dihydrate has previously been described in water and some organic media; however, the solvents for which published literature exists are those most commonly used in inhalation drug development, such as various ionic and ethanol solutions, not the organic solvents most commonly used in the preparation of polymeric nanoparticles. The solubility of the fumarate salt of formoterol has a water solubility of 1.16 ± 0.02 mg/mL at 25 °C. Solubility is increased with the increasing volume fraction of low molecular weight alcohols such as ethanol and methanol (Figure 1A). Similar to previously reported studies, formoterol fumarate dihydrate likely forms a less soluble solvate with ethanol at volume fractions greater than 50% and sees a subsequent reduction in solubility with increasing cosolvent fraction. Similar solvent formation was not seen in water–methanol mixtures; however, this has been previously reported under different experimental conditions. Formoterol fumarate dihydrate sees increasing solubility in highly basic or acidic conditions (Figure 1B) as formoterol fumarate dihydrate contains both acidic and basic pKa(s) of around 8.6 and 9.8, respectively. Solubility of formoterol fumarate dihydrate in common non-ionic surfactants polyvinyl alcohol (PVA) and Pluronic^®^ F127 remains unchanged at concentrations ranging from 0.1% to 5% in aqueous solution. Solubilization is increased, however, in a concentration-dependent manner above the critical micelle concentration of sodium cholate (12 mM or 0.52%), an ionic surfactant (Figure 1C). Solubility in the common organic solvents dichloromethane (DCM), chloroform, acetonitrile, and acetate are also reported (Table 1), with acetone having the greatest solubility of 0.063 ± 0.004 mg/mL.

### 3.2. Effect of Polymeric Nanoparticle Synthesis Parameters on Formoterol Drug Loading

**Figure 2:**
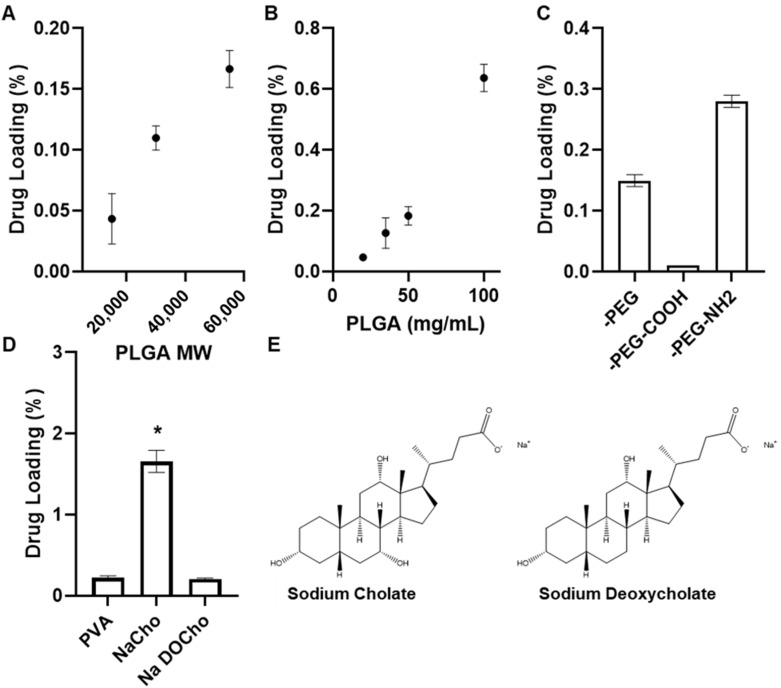
Formoterol drug loading of nanoparticles (**A**) prepared by single emulsion with increasing PLGA molecular weight, (**B**) prepared by single emulsion with increasing PLGA concentration, (**C**) prepared by double emulsion with modified PEG groups, (**D**) prepared by double emulsion with alterations to inner aqueous phase surfactant (**E**) structures of sodium cholate and sodium deoxycholate. PVA; 1% polyvinyl alcohol, NaCho; 12 mM sodium cholate, NaDOChol; 10 mM sodium deoxycholate. * indicates significance (*p* < 0.05). All data are presented as mean (*n* = 3) ± s.d.

Achieving significant drug loading of formoterol in PLGA nanoparticles is complicated by the low solubility in organic media, such as DCM and acetonitrile, where PLGA is freely soluble, compared to low molecular weight alcohols such as methanol, where PLGA and PLGA conjugates are practically insoluble. Drug loading of formoterol in PLGA-PEG nanoparticles prepared by single emulsion is improved with increasing PLGA molecular weight (35 mg/mL PLGA-PEG held constant), from 0.04% to 0.17% (Figure 2A), and increasing PLGA-PEG concentration (using 55,000 MW PLGA-PEG), up to 0.63% (Figure 2B). This was the maximum drug loading achievable by a single emulsion solvent evaporation method. While the higher molecular weight polymer (55,000 MW PLGA) achieved increased drug loading, the increases in drug loading are offset by increased nanoparticle size (data not shown) and reportedly increased degradation time in vivo [3,4,5], which could potentially lead to accumulation and toxicity. For these reasons, 20,000 MW PLGA polymers were selected for further study. 

Formoterol loading is further enhanced by changing from a single to double emulsion method and modification of the PEG terminus. Nanoparticles were prepared by water-in-oil-in-water double emulsion using PLGA-PEG, carboxylic acid modified PEG, or amine-modified PEG. PLGA-PEG-COOH reduced drug loading compared to methyl terminated PEG from 0.15% to 0.01%, whereas PLGA-PEG-NH_2_ significantly increased drug loading to 1.39% (Figure 2C). 

Interaction between the formoterol and the inner aqueous surfactant was evaluated using 10 mg/mL 20,000 MW PLGA-PEG-NH_2_ and either 1% PVA, 12 mM sodium cholate or 10 mM sodium deoxycholate as the inner phase. The use of 10 mM sodium deoxycholate was required as stable nanoparticles did not form using 12 mM sodium deoxycholate. The use of the nonionic homopolymer surfactant PVA as the inner phase showed increased (0.22%) drug loading compared to their equivalent single emulsion prepared particles (Figure 2D). However, the use of the ionic surfactant sodium cholate showed a significant increase in drug loading, up to 1.66%. This increase in loading is completely nullified by the use of 10 mM sodium deoxycholate, which differs from sodium cholate by only the 7α-hydroxyl group (structures Figure 2E), suggesting this interaction is critical to the improved loading seen by sodium cholate.

### 3.3. Effect of Polymeric Nanoparticle Synthesis Parameters on Particle Size

**Figure 3:**
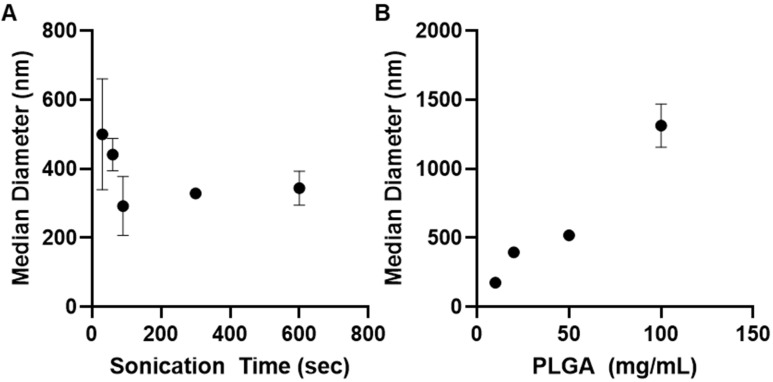
Median hydrodynamic diameter determined by zetasizer for nanoparticles prepared by double emulsion with (**A**) increasing 60 W ultrasonication time and (**B**) increasing polymer concentration in the organic phase. All data are presented as mean (*n* = 3) ± s.d.

The impact of sonication and PGLA concentration on particle size was evaluated with the goal of achieving particles with median hydrodynamic diameters between 300 and 500 nm. Sonication time was the first parameter to be evaluated for double emulsion-prepared particles (10 mg/mL PLGA-PEG-NH_2_, 10 mM sodium cholate inner phase), increasing the secondary sonication from 30 to 600 s. Initially, there was a precipitous decrease in median diameter, from over 500 nm to 292 nm with 90 s of sonication. Further increases in sonication time resulted in no significant change in particle size (Figure 3A).

The impact of polymer concentration was additionally determined at concentrations of PLGA-PEG-NH_2_ ranging from 10 to 100 mg/mL. All particles were prepared by double-emulsion, used 12 mM sodium cholate as an inner phase, and were sonicated for 180 s during preparation of the secondary emulsion. Particle size proved to be extremely sensitive to increasing PLGA concentration, with particle size increasing proportionally to the increase in polymer (Figure 3B).

**Figure 4:**
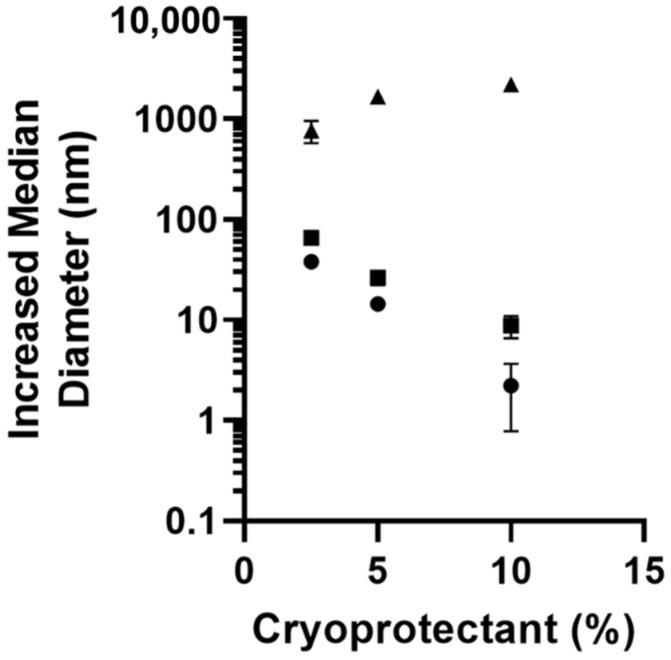
Median hydrodynamic diameter determined by zetasizer for PLGA-PEG-NH_2_ nanoparticles prepared by double emulsion using either 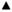 mannitol, 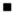 trehalose, or 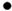 sucrose as cryoprotectants. All data are presented as mean (*n* = 3) ± s.d.

The impact of lyophilization was assessed following purification of the nanoparticles. To determine the impact of cryoprotectant selection and concentration on primary particle size, 1 mL of double-emulsion (12 mM sodium cholate inner phase, 180 s sonication) prepared PLGA-PEG-NH_2_ nanoparticles at a concentration of 10 mg/mL were lyophilized in either 2.5%, 5%, or 10% of either sucrose, trehalose, or d-mannitol for 72 h. Resuspended nanoparticles lyophilized with mannitol as the cryoprotectant showed significantly increased particle size, over 1 μm (Figure 4). Conversely, both sucrose and trehalose cryoprotectants demonstrated decreased particle size growth post lyophilization with increasing cryoprotectant concentration, with sucrose performing slightly better than trehalose at all concentrations. For further characterization studies using nanoparticles lyophilized with cryoprotectants, 5% cryoprotectant concentration was used.

### 3.4. Impact of Nanoparticle Synthesis Parameters on Zeta Potential

**Figure 5:**
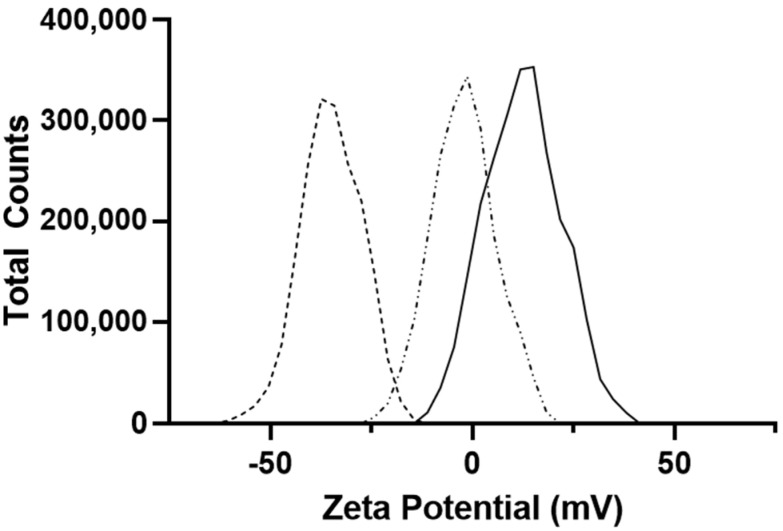
Zeta potential measurements of nanoparticles prepared with various PEG modifications immediately following synthesis after resuspension in 0.1× normal saline solution at 25 °C and pH 7.2. 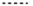 PLGA-PEG-COOH, 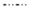 PLGA-PEG, 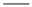 PLGA-PEG-NH_2_. All data are presented as mean (*n* = 3) ± s.d.

Nanoparticle zeta potential following washing was determined in 0.1× normal saline at 25 °C and neutral pH. Nanoparticle zeta potential was most strongly influenced by modification of PEG group (Figure 5). Methyl-endcapped PEG had a zeta potential of −0.792 ± 0.284 mV, whereas amine modified PEG had a positive zeta potential of 14.133 ± 0.404 mV and carboxylic acid modified PEG had a negative zeta potential of −34.87 ± 0.945 mV.

### 3.5. Impact of Nanoparticle Synthesis Parameters on Drug Release

**Figure 6:**
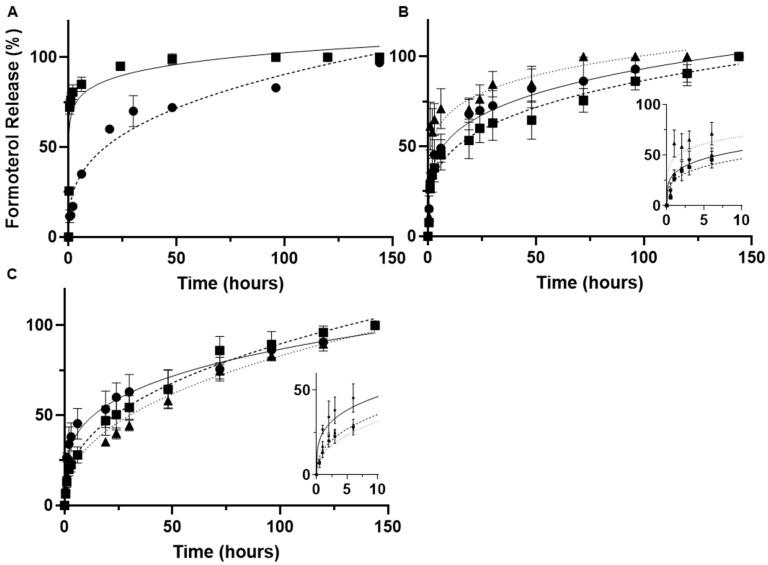
Cumulative formoterol release from nanoparticles at 37 °C and constant stirring (**A**) 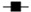 oil-in-water single emulsion 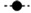 water-in-oil-in-water double emulsion (**B**) nanoparticles prepared by double emulsion with 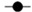 1% PVA inner phase, 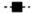 12 mM sodium cholate inner phase, 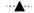 10 mM sodium deoxycholate inner phase (**C**) nanoparticles prepared by double emulsion using 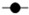 10 mg/mL, 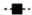 20 mg/mL, 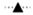 50 mg/mL PLGA-PEG-NH_2_. Inserts of the first 10 h are included for (**B**,**C**). All data are presented as the mean of (*n* = 3) ± s.d.

Formoterol release is significantly altered by the method of synthesis. Single and double emulsion solvent evaporation methods were assessed using 50 mg/mL 55,000 MW PLGA-PEG, 180 s sonication time and using 1% PVA as the inner aqueous phase. Release was measured out to 1 week (144 h) in PBS at 37 °C with constant stirring. Nanoparticles prepared by single emulsion showed significant burst release, with 80 and 90% of their entrapped drug released within the first 3 and 24 h, respectively. Comparatively, nanoparticles prepared by double emulsion demonstrated a slower initial phase of release, with 3-h release at 17% and 24-h release between 60 and 70% (Figure 6A). Analysis of the release kinetics showed non-fickian/anomalous diffusion (n > 0.43) for single emulsion-prepared nanoparticles and quasi-fickian diffusion for (n < 0.43) for double emulsion-prepared nanoparticles (Table 2).

Modification of the inner phase surfactant also impacted the initial release. Nanoparticle were prepared using 10 mg/mL 20,000 MW PLGA-PEG-NH_2_ and by varying the inner phase between 1% PVA, 12 mM sodium cholate or 10 mM sodium deoxycholate. The PVA and sodium cholate-prepared particles showed slight differences in 3-h burse release (45 and 38%, respectively) and both were significantly lower than the sodium deoxycholate-prepared particles, which showed 65% drug release by 3-h (Figure 6B). Analysis of release kinetics showed no significant difference in release exponent between PVA and sodium cholate inner phases; however, there was a significant (*p* < 0.05) difference between those and sodium deoxycholate (Table 2). These differences were less apparent during the sustained release phase (time > 24 h). Finally, increasing concentration of PLGA-PEG-NH_2_ during nanoparticle synthesis using 12 mM sodium cholate as the inner phase resulted in a decreased percentage of formoterol released within the first 3 h and increased rate of release beyond 24 h (Figure 6C). Increasing polymer concentration resulted in increasing the release exponent towards fickian (n = 0.43) release (Table 2).

### 3.6. Characterization of Nanoparticle Surface Morphology

**Figure 7:**
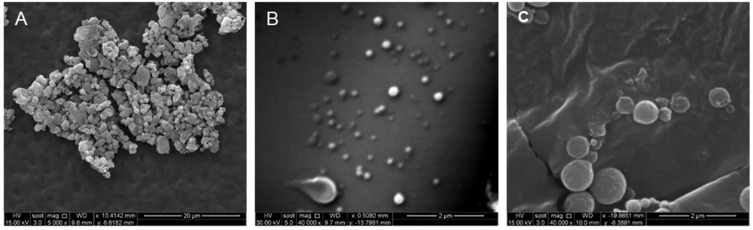
Scanning electron micrographs taken of double emulsion prepared nanoparticles of (**A**) PLGA-PEG, (**B**) PLGA-PEG-COOH, (**C**) PLGA-PEG-NH_2_.

The surface characteristics of nanoparticles prepared by double emulsion were assessed. Nanoparticles of PLGA-PEG with sodium cholate inner phase showed a high degree of surface roughness and size irregularity as well as a tendency to agglomerate (Figure 7A). Nanoparticles prepared by double-emulsion with PLGA-PEG-COOH and PLGA-PEG-NH_2_ with sodium cholate inner phase were similarly sized, producing spherical particles that did not form aggregates and had smooth surface features (Figure 7B,C).

### 3.7. X-ray Powder Diffraction (XRPD)

**Figure 8:**
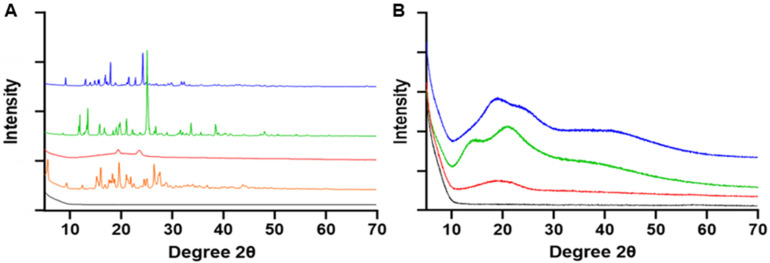
XRPD diffractograms (**A**) formoterol fumarate dihydrate (orange), PLGA-PEG-NH_2_ (red), sucrose (green), and trehalose (blue) and (**B**) nanoparticles lyophilized without cryoprotectant (red), nanoparticles lyophilized with either sucrose (green) or trehalose (blue) cryoprotectants. Black is the blank background.

X-ray diffractograms of nanoparticle raw materials (PLGA-PEG-NH_2_, formoterol fumarate dihydrate and cryoprotectants) as well as lyophilized nanoparticles with and without cryoprotection were obtained (Figure 8). The diffraction pattern of raw materials formoterol fumarate dihydrate, sucrose, and trehalose showed multiple sharp peaks across the scanned range, indicating long range molecular order consistent with crystallinity (Figure 8A). Raw polymer samples and all lyophilized-prepared nanoparticles did not contain any sharp crystalline peaks (Figure 8A,B).

### 3.8. Thermal Analysis of Lyophilized Nanoparticles

Thermal analysis of nanoparticle components formoterol fumarate dihydrate, PLGA-PEG- NH_2_, sucrose and trehalose as well as lyophilized nanoparticles with or without cryoprotectants are summarized in (Table 3).

**Figure 9:**
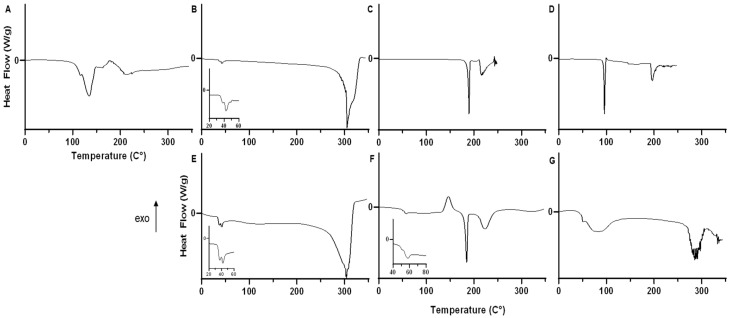
Differential scanning calorimetry thermograms of (**A**) formoterol fumarate dihydrate, (**B**) PLGA-PEG-NH_2_ (**C**) Sucrose, (**D**) Trehalose, (**E**) PLGA-PEG-NH_2_ nanoparticles lyophilized without cryoprotectant, (**F**) nanoparticles lyophilized with 5% sucrose cryoprotectant, (**G**) nanoparticles lyophilized with 5% trehalose cryoprotectant.

For formoterol fumarate dihydrate, there was a bimodal endotherm with an initial peak of 104 °C and a main peak of 130 °C. Above 150 °C, thermal decomposition was seen in the form of a jagged baseline (Figure 9A). For PLGA-PEG-NH_2_ polymer, there was a clear glass transition peak (T_g_) from 1–43 °C combined with an endotherm at 48 °C and a broad decomposition starting at 250 °C (Figure 9B). Raw sucrose showed a sharp endotherm at 189 °C, followed by a broad decomposition endotherm at 220 °C (Figure 9C). Trehalose dihydrate showed a sharp endotherm at 95 °C, followed by a broad endotherm at 193 °C, followed by decomposition (Figure 9D). PLGA-PEG-NH_2_ particles lyophilized without cryoprotectant showed slightly decreased T_g_ of 35–36 °C compared to the raw polymer and a similar first endotherm at 44 °C, followed by a broad endotherm starting from 82 °C and peaking at 112 °C (Figure 9E). Nanoparticles lyophilized with 5% sucrose as a cryoprotectant showed an increased T_g_ and first endotherm comparted to the raw polymer, 50–54 °C and 57 °C, respectively. Additionally, the broad endotherm at 96 °C transitioned into an exothermic peak at 148 °C, followed by an endotherm at 184 °C and secondary endotherm at 222 °C leading to decomposition (Figure 9F). Finally, nanoparticles lyophilized with trehalose as a cryoprotectant had a similar T_g_ to the raw polymer, 43–46 °C followed by a broad endotherm starting from 58 °C and peaking at 86 °C. No other endotherms were detected until decomposition started above 250 °C.

**Figure 10:**
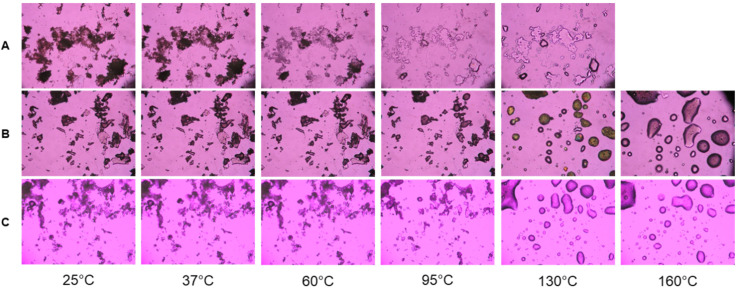
Representative hot stage microscopy images for (**A**) PLGA-PEG-NH_2_ nanoparticles lyophilized without cryoprotectant, (**B**) nanoparticles lyophilized with 5% sucrose cryoprotectant, (**C**) nanoparticles lyophilized with 5% trehalose cryoprotectant.

Lyophilized nanoparticles were also evaluated by cross-polarized HSM. All three particles (lyophilized without cryoprotectant, with sucrose cryoprotectant and with trehalose cryoprotectant) were dark and lacked birefringence at 25 °C and 37 °C. At 60 °C, the cryoprotectant free particles began melting, a process that continued until melting was fully completed at 130 °C (Figure 10A). Both formulations lyophilized with cryoprotectants did not have observable melts until closer to 100 °C (Figure 10B,C). Nanoparticles lyophilized with sucrose exhibited a liquid crystal transition, as noted by the marked diffuse birefringence at 130 °C (Figure 10B). Both lyophilates had completely melted by 160 °C and there were no observable transitions after that temperature.

### 3.9. Water Content Analysis by Karl Fischer Titration

Water content was determined for double emulsion-prepared lyophilates with and without cryoprotection (Table 4). For lyophilates of PLGA-PEG without cryoprotection, water content was 1.38 ± 0.20%. Comparatively, amine modification of the PEG group increased water content to 2.20 ± 0.61%. Lyophilization with 5% of either sucrose or trehalose resulted in significantly (*p* < 0.05) reduced water content, 0.78 ± 0.17% and 0.80 ± 0.19%, respectively.

## 4. Discussion

Formoterol fumarate dihydrate is an FDA-approved long-acting beta-2 adrenergic agonist that has been approved for the treatment of asthma and chronic obstructive pulmonary disease [42,43] and has shown promise in treating mitochondrial dysfunction in a variety of diseases [14,15,16,18,20,21]. As a raw material it exists as a crystalline powder that has been reported to be slightly soluble in water, soluble and sparingly soluble in methanol and ethanol, respectively, and practically insoluble in acetone and diethyl ether. The objective of this study was to develop a method of entrapping formoterol within a polymeric nanoparticle 300–500 nm in diameter for sustained drug release. Initially, an evaluation of the solubility of formoterol fumarate dihydrate (FFD) was required to determine optimal nanoparticle preparation, given FFD is only slightly soluble in water (1.16 ± 0.02 mg/mL) (Figure 1), which is in good agreement with the published literature [41]. Ethanol/Methanol and water mixtures show exponentially increasing solubilities of FFD with increasing alcohol content (Figure 1A). However, above 50% volume fraction of ethanol there is a decrease in solubility likely through the formation of an ehtanolate, which has been described previously [41]. Solubility in common organics used in nanoparticle syntheses was also established as low, with FFD being practically insoluble in dichloromethane (DCM), chloroform, acetonitrile, and acetone (Table 1). Solubility in nonionic surfactants including the homopolymer PVA and the triblock copolymer Pluronic F127 was unchanged; however, it increased significantly with the addition of ionic surfactant sodium cholate, with solubility increasing above the critical micelle concentration (12 mM or 0.5%) (Figure 1C). Given the apparent amphiphilic nature of FFD, both single (oil-in-water) and double (water-in-oil-in-water) methods of nanoparticle synthesis were evaluated.

Achieving significant drug loading by single emulsion solvent evaporation methods was challenging. Due to the low solubility of FFD in the common solvents DCM and chloroform, methanol, selected due to the decreased likelihood of forming solvates with formoterol, was added to allow sufficient FFD concentrations in the organic phase. Increased drug loading was observed with increased molecular weight of PLGA as well as increasing PLGA-PEG concentration in the organic phase (Figure 2A,B), both of which are in agreement with previously reported trends in loading of hydrophobic drugs [44,45,46]. Increasing PLGA concentration had a measured impact on nanoparticle size as well, with increasing polymer concentration resulting in increasing median hydrodynamic diameter **(**Figure 3B), which has also been previously reported for single emulsion prepared nanoparticles [46,47].

Sonication time was additionally optimized, with median particle diameter being reduced with increasing sonication time up to 90 s, and no significant change in median diameter with additional sonication time (Figure 3A).

Single emulsion nanoparticles were prepared; however, they exhibited significant burst release (Figure 6A), with over 90% of the entrapped drug being released within the first 24 h. This, along with first-order release kinetics (Table 2), suggests that in the PLGA-PEG single emulsion-prepared particles, the formoterol was not well entrapped within the polymer matrix but rather resided predominantly on the PLGA-PEG surface and the formoterol release was primarily diffusion limited.

In an effort to improve drug loading and the slow release of formoterol, a double emulsion solvent evaporation method of preparing nanoparticles was evaluated along with modifications to the polymer. By modifying the PEG group with either terminal acidic or amine residues. Nanoparticles prepared using 10 mg/mL polymer and a 1% PVA inner phase were compared and showed significantly improved (*p* < 0.05) drug loading with the amine modified polymer compared to both methyl and acidic residue terminated PEG. This was potentially due to the amine residues on the surface and microdomains, described by Rabanel et al. (2014) [48], within the polymer matrix interacting with formoterol and preventing its diffusion out of the organic phase as the polymer hardened. The modifications to the PEG surface moieties also had the effect of modifying the zeta potential of the prepared particles, with amine terminated PEG particles having a more positive zeta potential and carboxylic acid-terminated PEG particles having a more negative zeta potential (Figure 5). The impact of greater (more non-zero) surface potential is likely greater physical stability in suspension as the particles with higher surface charges are less likely to form agglomerates [49,50,51].

Further modification to the double emulsion method by modifying the inner phase was accomplished by exchanging the 1% PVA for a 0.5% solution of sodium cholate, which significantly increased drug loading (Figure 2D). The increase in loading is not likely due to any increased viscosity of the sodium cholate solution, which has a literature value (0.90–0.91 mPa s [52]) of approximately half that of a 1% PVA solution (1.6–2.5 mPa s [53]) at 25 °C. The improved drug loading was completely ameliorated when the sodium cholate was switched for sodium deoxycholate at the same concentration. The 7α-hydroxyl group of sodium cholate, critical in forming a hydrophilic axis in sodium cholate secondary structures [54,55], was not present in the sodium deoxycholate (Figure 2E), suggesting that interactions with this hydrophilic moiety were responsible for the increased loading. The addition of sodium cholate also appears to slow formoterol release. Nanoparticles prepared with sodium cholate have a significantly (*p* < 0.05) slower release than those made with sodium deoxycholate (Figure 6B, Table 2). However, there was no significant difference between particles prepared with either 0.5% sodium cholate or 1% PVA inner phases. Lastly, there was no significant impact on drug release kinetics from increasing amine-terminated PEG polymer concentration in double emulsion-prepared nanoparticles (Figure 6C). However, there is a clear trend between increasing Fickian release character and increasing polymer concentration, with n trending towards 0.43 (Table 2). Differences in drug loading, especially between single and double emulsion-prepared particles, would also impact release kinetics as greater concentration gradients would directly impact diffusion limited release mechanisms.

Selecting a suitable means of lyophilization is a critical and often overlooked aspect of nanoparticle synthesis methods, as the changes in solid state physicochemical properties can have significant impacts on particle size, stability, and drug release [56,57,58]. In this study, we assessed the impact of increasing concentrations of three cryoprotectants: sucrose, trehalose, and mannitol. Mannitol had a significantly detrimental impact on primary particle size, with median hydrodynamic diameter increasing ~1 µm over the range tested (Figure 4). Sucrose and trehalose cryoprotectants were much improved, with changes in nanoparticle size decreasing with increased cryoprotectant. Both sucrose and trehalose saw less than 30 nm increases in particle size at 5% cryoprotectant (Figure 4).

The impact of nanoparticle synthesis and lyophilization is apparent on powder analysis. With raw FFD, bound water is initially removed in an endotherm preceding the main melting endotherm at 130 °C (Figure 9A), which is in good agreement with the literature [41]. This melting is not apparent in any of the prepared nanoparticles (Figure 9E–G), potentially due to formoterol being in an amorphous state [40,41]. This is further supported by the lack of sharp peaks in the XRPD patterns (Figure 8) of the lyophilized nanoparticles, which indicates no long-range molecular order and is consistent with an amorphous particle. Comparing the glass transition temperatures (T_g_) of raw PLGA-PEG-NH_2_ polymer to that of lyophilized nanoparticles without cryoprotectant shows a ~6 °C decrease in T_g_, which is potentially due to greater adsorbed water in the lyophilized particles (Table 4). Cryoprotection with either sucrose or trehalose showed no significant change in polymer glass transition (Figure 9F,G) and resulted in higher initial endotherm, which is attributed to the melting of PEG groups, and is visible in HSM micrographs (Figure 10). Cryoprotection of nanoparticles with sucrose results in an exotherm not present in the raw sucrose, a recrystallization peak at 148 °C (Figure 9F), which is confirmed by birefringency above 130 °C in HSM micrographs (Figure 10B), as has been previously described following sucrose lyophilization [59,60]. Lyophilization of nanoparticles with trehalose produces no similar amorphous-to-crystalline transition, only a broad melting endotherm between 60 and 120 °C (Figure 9G and Figure 10C). Increased adsorbed water and decreased T_g_, as indicated in nanoparticles lyophilized without cryoprotection, could negatively impact the solid-state stability of nanoparticles as well as impact the drug release [61,62,63,64,65], further reinforcing the importance of cryoprotection when lyophilizing nanoparticles. In these experiments, as stated in the Methods section, the prepared and lyophilized nanoparticles are stored at −20 °C until use. Under these conditions, it is highly unlikely that chemical degradation of the formoterol or polymer would occur. Additionally, the lowered molecular mobility of the polymer and entrapped drug at these temperatures makes spontaneous release of the drug highly unlikely as well.

## 5. Conclusions

This comprehensive and systematic study focused on the design, development, characterization, and in vitro drug release of PEGylated PLGA polymeric nanoparticles containing formoterol drug for sustained release drug delivery applications. Initial formoterol drug loading, nanoparticle size, and in vitro drug release kinetics of the polymeric nanoparticles were improved upon by modification of synthesis parameters such as polymer molecular weight, polymer concentration, PEG modification, surfactant selection, and sonication time. These changes resulted in significantly improved drug loading and sustained release over the course of 7 days. Solid state characterization of the nanoparticles lyophilates has also been reported and shows the importance of cryoprotectant selection in preserving nanoparticle characteristics.

## Figures and Tables

**Table 1:** Solubility of formoterol in various organic solvents. DCM; dichloromethane, ACN; acetonitrile. Data are presented as the mean (*n* = 3) ± s.d.

Solvent	Formoterol Solubility (mg/mL)
DCM	0.001 ± 0.0004
Chloroform	0.002 ± 0.001
ACN	0.005 ± 0.001
Acetone	0.051 ± 0.004

**Table 2:** Nanoparticle release kinetics modeling. o/w; oil-in-water single emulsion w/o/w; water-in-oil-in-water double emulsion, PVA; nanoparticles prepared by double emulsion using 1% PVA inner phase, NaCholate; nanoparticles prepared by double emulsion using 12 mM sodium cholate inner phase, NaDOCholate; nanoparticles prepared by double emulsion using 10 mM sodium deoxycholate inner phase, 10/20/50 mg/mL; nanoparticles prepared by double emulsion using 10/20/50 mg/mL of polymer in the organic phase. All data are presented as the mean of (*n* = 3) release profiles.

	Zero Order	First Order	Korsmeyer-Peppas	
	R^2^	R^2^	R^2^	n
o/w	0.33	0.94	0.88	0.56
w/o/w	0.77	0.97	0.97	0.34
PVA	0.65	0.89	0.94	0.23
NaCholate	0.75	0.86	0.94	0.27
NaDOCholate	0.49	0.81	0.84	0.17
10 mg/mL	0.75	0.86	0.94	0.27
20 mg/mL	0.85	0.96	0.97	0.40
50 mg/mL	0.92	0.96	0.98	0.42

**Table 3:** Differential scanning calorimetry (DSC) thermal analysis. T_g_; glass transition temperature. Data presented are mean (*n* = 3) ± s.d.

	Tg (°C)			∆Cp	Endotherm #1			Endotherm #2			Endotherm #3			Endotherm #4			Exotherm #1		
Sample	onset (°C)	mid (°C)	end (°C)	J/g °C	onset (°C)	peak (°C)	enthalpy (J/g)	onset (°C)	peak (°C)	enthalpy (J/g)	onset (°C)	peak (°C)	enthalpy (J/g)	onset (°C)	peak (°C)	enthalpy (J/g)	onset (°C)	peak (°C)	enthalpy (J/g)
Raw Formoterol Fumarate Dihydrate					88.84 ± 1.87	104.62 ± 3.62	13.56 ± 1.04	116.28 ± 0.76	130.82 ± 2.73	136.7 ± 0.89									
Raw PLGA-PEG-NH2	41.56 ± 1.34	42.43 ± 1.51	43.34 ± 1.93	0.69 ± 0.44	43.32 ± 2.16	47.97 ± 4.72	4.95 ± 3.34												
Raw Sucrose											187.01 ± 0.59	189.43 ± 0.23	129.47 ± 0.4	219.35 ± 5.56	220.72 ± 4.48	150 ± 50.01			
Raw Trehalose								94.46 ± 0.07	95.79 ± 0.05	94.54 ± 2.28	190.98 ± 1.75	193.3 ± 3.24	92.98 ± 14.66						
Nanoparticle No Cryoprotectant	35.3 ± 1.25	36.12 ± 0.8	36.48 ± 0.69	0.3 ± 0.2	42.16 ± 6.75	43.99 ± 6.73	1.98 ± 1.14	82.62 ± 2.34	112.95 ± 9.6	8.67 ± 3.24									
Nanoparticle Sucrose Cryoprotectant	50.09 ± 1.1	54.3 ± 0.21	54.82 ± 0.43	1.34 ± 0.38	53.45 ± 0.32	57.15 ± 0.33	4.13 ± 0.43	70.93 ± 2.3	96.02 ± 1.58	10.11 ± 0.56	180.03 ± 0.36	184.74 ± 0.03	86.59 ± 2.38	213.08 ± 5.68	222.78 ± 2.12	119.77 ± 1.16	136.56 ± 1.93	148.26 ± 3.06	79.36 ± 5.03
Nanoparticle a Trehalose Cryoprotectant	43.08 ± 3.39	44.68 ± 4.02	46.64 ± 2.94	1.21 ± 0.31	42.97 ± 1.61	50.17 ± 0.18	4.73 ± 3.73	58.99 ± 1.99	86.75 ± 3.45	16.89 ± 2.72									

**Table 4:** Residual water content of lyophilized nanoparticles with and without cryoprotectants. * indicates significantly different from other groups (*p* < 0.05) Data are presented as the mean (*n* = 3).

Nanoparticle	Water% (*w*/*w*)
PLGA-PEG (no cryoprotectant)	1.38 ± 0.20
PLGA-PEG-NH_2_ (no cryoprotectant)	2.20 ± 0.61
PLGA-PEG-NH_2_ (sucrose)	0.78 ± 0.17 *
PLGA-PEG-NH_2_ (trehalose)	0.80 ± 0.19 *

## Data Availability

The data presented in this study are available on request from the Corresponding Author.

## References

[1] Couvreur P. (2013). Nanoparticles in drug delivery: Past, present and future. Adv. Drug Deliv. Rev..

[2] Farokhzad O.C., Langer R. (2009). Impact of nanotechnology on drug delivery. ACS Nano.

[3] Chenthamara D., Subramaniam S., Ramakrishnan S.G., Krishnaswamy S., Essa M.M., Lin F.-H., Qoronfleh M.W. (2019). Therapeutic efficacy of nanoparticles and routes of administration. Biomater. Res..

[4] Mansour H.M., Sohn M., Al-Ghananeem A.P.P. (2010). Materials for Pharmaceutical Dosage Forms: Molecular Pharmaceutics and Controlled Release Drug Delivery Aspects. Int. J. Mol. Sci..

[5] Rhee Y.S., Park C.W., DeLuca P.P., Mansour H.M. (2010). Sustained-Release Injectable Drug Delivery Systems. Pharm. Technol. Spec. Issue-Drug Deliv..

[6] Moghimi S.M., Hunter A.C., Murray J.C. (2001). Long-circulating and target-specific nanoparticles: Theory to practice. Pharmacol. Rev..

[7] Williams R.M., Shah J., Tian H.S., Chen X., Geissmann F., Jaimes E.A., Heller D.A. (2018). Selective Nanoparticle Targeting of the Renal Tubules. Hypertension.

[8] Muralidharan P., Mallory E., Malapit M., Hayes D., Mansour H.M. (2014). Inhalable PEGylated Phospholipid Nanocarriers and PEGylated Therapeutics for Respiratory Delivery as Aerosolized Colloidal Dispersions and Dry Powder Inhalers. Pharmaceutics.

[9] Williams R.M., Shah J., Ng B.D., Minton D.R., Gudas L.J., Park C.Y., Heller D.A. (2015). Mesoscale nanoparticles selectively target the renal proximal tubule epithelium. Nano Lett..

[10] Williams R.M., Jaimes E.A., Heller D.A. (2016). Nanomedicines for kidney diseases. Kidney Int..

[11] Han S.J., Williams R.M., D’Agati V., Jaimes E.A., Heller D.A., Lee H.T. (2020). Selective nanoparticle-mediated targeting of renal tubular Toll-like receptor 9 attenuates ischemic acute kidney injury. Kidney Int..

[12] Kim S.H., Jeong J.H., Chun K.W., Park T.G. (2005). Target-specific cellular uptake of PLGA nanoparticles coated with poly(L-lysine)-poly(ethylene glycol)-folate conjugate. Langmuir.

[13] Beck-Broichsitter M., Gauss J., Gessler T., Seeger W., Kissel T., Schmehl T. (2010). Pulmonary targeting with biodegradable salbutamol-loaded nanoparticles. J. Aerosol. Med. Pulm. Drug Deliv..

[14] Jesinkey S.R., Funk J.A., Stallons L.J., Wills L.P., Megyesi J.K., Beeson C.C., Schnellmann R.G. (2014). Formoterol restores mitochondrial and renal function after ischemia-reperfusion injury. J. Am. Soc. Nephrol..

[15] Cleveland K.H., Brosius F.C., Schnellmann R.G. (2020). Regulation of mitochondrial dynamics and energetics in the diabetic renal proximal tubule by the β. Am. J. Physiol. Renal. Physiol..

[16] Scholpa N.E., Williams H.C., Wang W., Corum D., Narang A., Tomlinson S., Sullivan P., Rabchevsky A.S., Schnellmann R. (2019). Pharmacological Stimulation of Mitochondrial Biogenesis Using the Food and Drug Administration-Approved β. J. Neurotrauma.

[17] Scholpa N.E., Simmons E.C., Crossman J.D., Schnellmann R.G. (2021). Time-to-treatment window and cross-sex potential of Beta 2-adrenergic receptor-induced mitochondrial biogenesis-mediated recovery after spinal cord injury. Toxicol. Appl. Pharmacol..

[18] Arif E., Solanki A.K., Srivastava P., Rahman B., Fitzgibbon W.R., Deng P., Budisavljevic M.N., Baicu C.F., Zile M.R., Megyesi J. (2019). Mitochondrial biogenesis induced by the β2-adrenergic receptor agonist formoterol accelerates podocyte recovery from glomerular injury. Kidney Int..

[19] Vekaria H.J., Hubbard W.B., Scholpa N.E., Spry M.L., Gooch J.L., Prince S.J., Schnellmann R.G., Sullivan P.G. (2020). Formoterol, a beta-2-adrenoreceptor agonist, induces mitochondrial biogenesis and promotes cognitive recovery after traumatic brain injury. Neurobiol. Dis..

[20] Bhargava P., Schnellmann R.G. (2017). Mitochondrial energetics in the kidney. Nat. Rev. Nephrol..

[21] Cameron R.B., Gibbs W.S., Miller S.R., Dupre T.V., Megyesi J., Beeson C.C., Schnellmann R.G. (2019). Proximal Tubule Beta-2 Adrenergic Receptor Mediates Formoterol-Induced Recovery of Mitochondrial and Renal Function After Ischemia-Reperfusion Injury. J. Pharmacol. Exp. Ther..

[22] Levine M.A., Leenen F.H. (1989). Role of beta 1-receptors and vagal tone in cardiac inotropic and chronotropic responses to a beta 2-agonist in humans. Circulation.

[23] Brodde O.E. (1991). Beta 1- and beta 2-adrenoceptors in the human heart: Properties, function, and alterations in chronic heart failure. Pharmacol. Rev..

[24] Vyas F.S., Nelson C.P., Freeman F., Boocock D.J., Hargreaves A.J., Dickenson J.M. (2017). β 2-adrenoceptor-induced modulation of transglutaminase 2 transamidase activity in cardiomyoblasts. Eur. J. Pharmacol..

[25] Koziczak-Holbro M., Rigel D.F., Dumotier B., Sykes D.A., Tsao J., Nguyen N., Bösch J., Jourdain M., Flotte L., Adachi Y. (2019). Pharmacological Characterization of a Novel 5-Hydroxybenzothiazolone-Derived β 2-Adrenoceptor Agonist with Functional Selectivity for Anabolic Effects on Skeletal Muscle Resulting in a Wider Cardiovascular Safety Window in Preclinical Studies. J. Pharmacol. Exp. Ther..

[26] Molenaar P., Chen L., Parsonage W.A. (2006). Cardiac implications for the use of beta2-adrenoceptor agonists for the management of muscle wasting. Br. J. Pharmacol..

[27] Yin Q., Yang C., Wu J., Lu H., Zheng X., Zhang Y., Lv Z., Zheng X., Li Z. (2016). Downregulation of β-Adrenoceptors in Isoproterenol-Induced Cardiac Remodeling through HuR. PLoS ONE.

[28] Dorn G.W. (2002). Adrenergic pathways and left ventricular remodeling. J. Card. Fail..

[29] Brouri F., Findji L., Mediani O., Mougenot N., Hanoun N., le Naour G., Hamon M., Lechat P. (2002). Toxic cardiac effects of catecholamines: Role of beta-adrenoceptor downregulation. Eur. J. Pharmacol..

[30] He J., Chen H., Zhou W., Chen M., Yao Y., Zhang Z., Tan N. (2020). Kidney targeted delivery of asiatic acid using a FITC labeled renal tubular-targeting peptide modified PLGA-PEG system. Int. J. Pharm..

[31] Yu H., Lin T., Chen W., Cao W., Zhang C., Wang T., Ding M., Zhao S., Wei H., Guo H. (2019). Size and temporal-dependent efficacy of oltipraz-loaded PLGA nanoparticles for treatment of acute kidney injury and fibrosis. Biomaterials.

[32] Nair A.V., Keliher E.J., Core A.B., Brown D., Weissleder R. (2015). Characterizing the interactions of organic nanoparticles with renal epithelial cells in vivo. ACS Nano.

[33] Vallorz E.L., Blohm-Mangone K., Schnellmann R.G., Mansour H.M. (2021). Formoterol PLGA-PEG Nanoparticles Induce Mitochondrial Biogenesis in Renal Proximal Tubules. AAPS J..

[34] Aodah A., Pavlik A., Karlage K., Myrdal P.B. (2016). Preformulation Studies on Piperlongumine. PLoS ONE.

[35] Akapo S.O., Asif M. (2003). Validation of a RP-HPLC method for the assay of formoterol and its related substances in formoterol fumarate dihydrate drug substance. J. Pharm. Biomed. Anal..

[36] Zhang Z., Feng S.S. (2006). The drug encapsulation efficiency, in vitro drug release, cellular uptake and cytotoxicity of paclitaxel-loaded poly(lactide)-tocopheryl polyethylene glycol succinate nanoparticles. Biomaterials.

[37] Duan J., Mansour H.M., Zhang Y., Deng X., Chen Y., Wang J., Pan Y., Zhao J. (2012). Reversion of multidrug resistance by co-encapsulation of doxorubicin and curcumin in chitosan/poly(butyl cyanoacrylate) nanoparticles. Int. J. Pharm..

[38] Cheng J., Teply B.A., Sherifi I., Sung J., Luther G., Gu F.X., Levy-Nissenbaum E., Radovic-Moreno A.F., Langer R., Farokhzad O.C. (2007). Formulation of functionalized PLGA-PEG nanoparticles for in vivo targeted drug delivery. Biomaterials.

[39] Alabsi W., Al-Obeidi F.A., Polt R., Mansour H.M. (2020). Organic Solution Advanced Spray-Dried Microparticulate/Nanoparticulate Dry Powders of Lactomorphin for Respiratory Delivery: Physicochemical Characterization, In Vitro Aerosol Dispersion, and Cellular Studies. Pharmaceutics.

[40] Tajber L., Corrigan D.O., Corrigan O.I., Healy A.M. (2009). Spray drying of budesonide, formoterol fumarate and their composites—I. Physicochemical characterisation. Int. J. Pharm..

[41] Jarring K., Larsson T., Stensland B., Ymén I. (2006). Thermodynamic stability and crystal structures for polymorphs and solvates of formoterol fumarate. J. Pharm. Sci..

[42] Sharafkhaneh A., Mattewal A.S., Abraham V.M., Dronavalli G., Hanania N.A. (2010). Budesonide/formoterol combination in COPD: A US perspective. Int. J. Chron. Obstruct. Pulmon. Dis..

[43] Saari S.M., Vidgren M.T., Herrala J., Turjanmaa V.M., Koskinen M.O., Nieminen M.M. (2002). Possibilities of formoterol to enhance the peripheral lung deposition of the inhaled liposome corticosteroids. Respir. Med..

[44] Jyothi N.V., Prasanna P.M., Sakarkar S.N., Prabha K.S., Ramaiah P.S., Srawan G.Y. (2010). Microencapsulation techniques, factors influencing encapsulation efficiency. J. Microencapsul..

[45] Wischke C., Schwendeman S.P. (2008). Principles of encapsulating hydrophobic drugs in PLA/PLGA microparticles. Int. J. Pharm..

[46] Ravi S., Peh K.K., Darwis Y., Murthy B.K., Singh T.R., Mallikarjun C. (2008). Development and characterization of polymeric microspheres for controlled release protein loaded drug delivery system. Indian J. Pharm. Sci..

[47] Song X., Zhao Y., Hou S., Xu F., Zhao R., He J., Cai Z., Li Y., Chen Q. (2008). Dual agents loaded PLGA nanoparticles: Systematic study of particle size and drug entrapment efficiency. Eur. J. Pharm. Biopharm..

[48] Rabanel J.M., Hildgen P., Banquy X. (2014). Assessment of PEG on polymeric particles surface, a key step in drug carrier translation. J. Control. Release.

[49] Jacobs C., Müller R.H. (2002). Production and characterization of a budesonide nanosuspension for pulmonary administration. Pharm. Res..

[50] Feng S., Huang G. (2001). Effects of emulsifiers on the controlled release of paclitaxel (Taxol) from nanospheres of biodegradable polymers. J. Control. Release.

[51] Vega E., Gamisans F., García M.L., Chauvet A., Lacoulonche F., Egea M.A. (2008). PLGA nanospheres for the ocular delivery of flurbiprofen: Drug release and interactions. J. Pharm. Sci..

[52] Ting W., Tong-Chun B., Wei W., Jian-Jun Z., Cheng-Wen Z. (2008). Viscosity and activation parameters of viscous flow of sodium cholate aqueous solution. J. Mol. Liq..

[53] Mohsen-Nia M., Modarress H. (2012). Viscometric study of aqueous poly(vinyl alcohol) (PVA) solutions as a binder in adhesive formulations. J. Adhes. Sci. Technol..

[54] Santhanalakshmi J., Lakshmi G.S., Aswal V.K., Goyal P.S. (2001). Small-angle neutron scattering study of sodium cholate and sodium deoxycholate interacting micelles in aqueous medium. Proc. Indian Acad. Sci..

[55] Maslova V.A., Kiselev M.A. (2018). Structure of Sodium Cholate Micelles. Crystallogr. Rep..

[56] Abdelwahed W., Degobert G., Stainmesse S., Fessi H. (2006). Freeze-drying of nanoparticles: Formulation, process and storage considerations. Adv. Drug Deliv. Rev..

[57] Fonte P., Soares S., Sousa F., Costa A., Seabra V., Reis S., Sarmento B. (2014). Stability study perspective of the effect of freeze-drying using cryoprotectants on the structure of insulin loaded into PLGA nanoparticles. Biomacromolecules.

[58] Holzer M., Vogel V., Mäntele W., Schwartz D., Haase W., Langer K. (2009). Physico-chemical characterisation of PLGA nanoparticles after freeze-drying and storage. Eur. J. Pharm. Biopharm..

[59] Kedward C.J., MacNaughtan W., Mitchell J.R. (2000). Isothermal and non-isothermal crystallization in amorphous sucrose and lactose at low moisture contents. Carbohydr. Res..

[60] van Eerdenbrugh B., Froyen L., Martens J.A., Blaton N., Augustijns P., Brewster M., van den Mooter G. (2007). Characterization of physico-chemical properties and pharmaceutical performance of sucrose co-freeze-dried solid nanoparticulate powders of the anti-HIV agent loviride prepared by media milling. Int. J. Pharm..

[61] Alexis F. (2004). Factors affecting the degradation and drug-release mechanism of poly(lactic acid) and poly[(lactic acid)-co-(glycolic acid)]. Polym. Int..

[62] Kranz H., Ubrich N., Maincent P., Bodmeier R. (2000). Physicomechanical properties of biodegradable poly(D,L-lactide) and poly(D,L-lactide-co-glycolide) films in the dry and wet states. J. Pharm. Sci..

[63] Makadia H.K., Siegel S.J. (2011). Poly Lactic-co-Glycolic Acid (PLGA) as Biodegradable Controlled Drug Delivery Carrier. Polymers.

[64] Xu Y., Kim C.S., Saylor D.M., Koo D. (2017). Polymer degradation and drug delivery in PLGA-based drug-polymer applications: A review of experiments and theories. J. Biomed. Mater. Res. B Appl. Biomater..

[65] Wang B., Tchessalov S., Cicerone M.T., Warne N.W., Pikal M.J. (2009). Impact of sucrose level on storage stability of proteins in freeze-dried solids: II. Correlation of aggregation rate with protein structure and molecular mobility. J. Pharm. Sci..

